# Ultrasound microflow patterns help in distinguishing malignant from benign thyroid nodules

**DOI:** 10.1186/s40644-024-00663-1

**Published:** 2024-01-25

**Authors:** Wanying Li, Luying Gao, Yiyan Du, Ying Wang, Xiao Yang, Hongyan Wang, Jianchu Li

**Affiliations:** 1 Department of Ultrasound, State Key Laboratory of Complex Severe and Rare Diseases, Peking Union Medical College Hospital, Chinese Academy of Medical Sciences and Peking Union Medical College, Beijing, China; 2 Department of Ultrasound, Wuxi Branch of Ruijin Hospital, Jiangsu, China

**Keywords:** Thyroid nodule, Ultrasound, Superb microvascular imaging, Microflow characteristics

## Abstract

**Background:**

Vascular features are not commonly used to evaluate thyroid nodules by conventional ultrasound due to the low sensitivity. Superb Microvascular Imaging (SMI) is a new ultrasonic Doppler technology that specializes in depicting microvessels and low-speed flow. The objective of this study was to explore the value of microflow features on SMI in differentiating malignant from benign thyroid nodules.

**Methods:**

One hundred and seventy-seven adult patients with thyroid nodules in our center from October 2021 to June 2022 with available histopathological results were recruited, including 125 malignant nodules and 123 benign nodules. Preoperative ultrasound was performed using greyscale, Color Doppler Flow Imaging (CDFI), monochrome SMI (mSMI) and color SMI (cSMI). Vascular features such as flow richness, microflow distribution and microflow patterns of malignant thyroid nodules were compared with those of benign nodules.

**Results:**

Penetrating vessel ≥ 1 (82.4% in the malignant group vs. 30.9% in the benign group, *P* < 0.001), the crab claw-like pattern (64.0% vs. 10.6%, *P* < 0.001) and the root hair-like pattern (8.0% vs. 2.4%, *P* = 0.049) were common in malignant thyroid nodules, among which the crab claw-like pattern was an independent risk factor for malignant thyroid nodules. The wheel-like pattern (1.6% in the malignant group vs. 33.3% in the benign group, *P* < 0.001) and the arborescent pattern (0 vs. 19.5%, *P* < 0.001) were more likely to appear in benign nodules. The diagnostic specificities of the crab claw-like pattern and the root hair-like pattern for malignant thyroid nodules were 0.894, 0.976, and the positive predictive values were 0.860, 0.769. The diagnostic specificities of the wheel-like pattern and the arborescent pattern for benign thyroid nodules were 0.984, 1.000, and the positive predictive values were 0.953, 1.000.

**Conclusions:**

The crab claw-like pattern and the root hair-like pattern were microflow characteristics of malignant thyroid nodules. The wheel-like pattern and the arborescent pattern could help exclude the diagnosis of thyroid cancer.

## Background

Recent years have witnessed an increase in the diagnosis of thyroid nodules [1]. The prevalence of thyroid nodules detected by high-resolution ultrasound can reach 68% in adults [2]. However, only 10%-15% are malignant and need clinical intervention [3]. Therefore, it is important to distinguish malignant from benign thyroid nodules. Ultrasound is the preferred examination method for thyroid lesions. The American College of Radiology Thyroid Imaging Reporting and Data System (ACR TI-RADS) [4] is widely used to evaluate the thyroid nodules according to the ultrasonic composition, echogenicity, margin, shape and echogenic foci. However, the greyscale features of benign and malignant thyroid nodules often overlap [5, 6]. More and more scholars are pursuing new methods and techniques.

The occurrence, development, invasion and metastasis of tumors are highly dependent on angiogenesis [7]. Excessive epithelial cells and perithelial cells promote the construction and extension of neovessels of thyroid cancer [8]. Theoretically, malignant thyroid nodules can be identified by different vascular features. Some researchers believed that the evaluation of the morphology and structure of vessels might be important for the differential diagnosis of thyroid nodules [9]. However, Color Doppler Flow Imaging (CDFI) is of limited use since it cannot display microvessels less than 0.1 mm in diameter or the microflow at a velocity less than 1 mm/s [10] with large observer variance. Spectral Doppler can detect abnormal flow parameters of malignant thyroid nodules, but the sensitivity is low [11]. Contrast enhanced ultrasound (CEUS) can clearly show the microflow of nodules, which benefits the diagnosis [12]. However, CEUS is an invasive examination, and its use is restricted by contrast agent contraindications. In view of these, vascularity, an important ultrasound feature, has not been included in the guidelines for ultrasonic evaluation of thyroid nodules and is not widely used.

As the third generation of ultrasonic Doppler technology after CDFI and Power Doppler Flow Imaging (PDFI), Superb Microvascular Imaging (SMI) may greatly solve the above problems. It can preserve the tiny flow signals and remove the clutter with the new algorithm and developed wall filter. In addition, it is safer and more convenient for free of contrast agents. Our study aimed to explore the vascular features of benign and malignant thyroid nodules, including richness, size, morphology, distribution and patterns of microflow, and to sort out new ultrasound indices for the noninvasive evaluation of thyroid nodules on SMI.

## Methods

### Study population

This retrospective study was approved by the Institutional Review Board of Peking Union Medical College Hospital (protocol number K3557) and the written informed consent of all patients was obtained. From October 2021 to June 2022, 200 consecutive adult patients with thyroid nodules who planned to undergo greyscale and SMI ultrasound examination as well as thyroid surgery in our center were prospectively recruited. The inclusion criteria were (a) greyscale ultrasound could clearly show the thyroid nodules, (b) solid or predominantly solid nodules, (c) the total thyroidectomy or lobectomy was planned to be performed within one week after the ultrasound examination, and the histopathological results were available after the operation. 23 patients were excluded for the following reasons: [1] the vascularity detection of the nodules near the cervical arteries was influenced by the arterial pulsation (*n* = 12), (b) acoustic shadow of the coarse calcification at the anterior of the nodules covered the flow signals (*n* = 9); (c) patients had preoperative treatment like thermal ablation therapy (*n* = 2). If the patient had more than one thyroid nodule, all the nodules matching the criteria were included. Finally, 177 patients (248 thyroid nodules) were enrolled in this research.

### Imaging acquisition

One radiologist with 4 years of experience in ultrasound scanning performed the examinations using Aplio 900 (Canon Medical Systems Corp., Tokyo, Japan) equipped with a 5-18 MHz linear transducer. Patients were instructed to lie supine, tilt the head back to fully expose the examination area and breathe smoothly, avoiding swallowing as much as possible. All nodules were carefully scanned in transverse and longitudinal planes on greyscale first. Then the CDFI and SMI examinations were performed for vascular features with no extra pressure applied to the transducer to avoid vessel collapse. The region of interest included the entire nodule and approximately 3–5 mm of the surrounding parenchyma on CDFI and SMI. The color gain was set to the maximum to exactly suppress the background noise. CDFI scale was set < 6.5 cm/s, frame rate 15f/s, dynamic range 60 dB. SMI scale at 1.0–2.5 cm/s, frame rate 25-60f/s, dynamic range 60 dB. Every nodule was scanned on monochrome SMI (mSMI) and color SMI (cSMI) modes respectively. Since mSMI is more sensitive to microflows [13], it served as the main mode for evaluating the microflow features, with cSMI helping to distinguish microflow from calcification. Smart-3D was also built to show the three-dimensional microflow pattern based on cSMI and instruct the plane selection for the one with the richest flow signals, which was used for microflow features analysis. Spectral Doppler was used to confirm the real vessels when necessary. The largest transverse and longitudinal planes on greyscale of every nodule, cine clips on CDFI and SMI and planes with the richest blood flow were saved.

### Imaging analysis

Blinded to the clinical and pathological information of the patients, two radiologists with over 5 years of experience in thyroid ultrasound independently analyzed all the images and videos. One of the two radiologists reviewed again 6 months later still unaware of the above information. The size of nodules was defined as the largest diameter. The greyscale features were assessed according to the ACR TI-RADS [4]. Solid composition, hypoechogenicity or very hypoechogenicity, irregular margin or extrathyroidal extension, taller-than-wide shape and microcalcification were acknowledged as greyscale malignant features. The flow richness of nodules was evaluated based on Adler’s grading [14]. For grade 0, no blood flow was detected; one or two pixels containing flow was considered grade 1; grade 2 was assigned for a main vessel and/or several small vessels (less than 1 mm in diameter); grade 3 included 4 or more vessels. The penetrating vessel referred to the vessel going from the outside to the inside of the nodule [15].

Besides these, microflow features included morphology, size, distribution and pattern. Microflow morphology was classified as dot-like, linear and tortuous. “Dot-like” meant that all microflows were dot-like on SMI. If over half of the microflows in one nodule were linear or tortuous, it was defined as “linear” or “tortuous”, respectively. The microflow size categories were dot-like, tiny and bulky. The “dot-like” was defined in the same way as that in microflow morphology. If over half of the microflows in one nodule were observed only on SMI, it was defined as “tiny”. And “bulky” was given when over half of the microflows in one nodule could be observed on both CDFI and SMI. The distribution of microflows was divided into five types: avascularity, peripheral vascularity, mainly peripheral vascularity, mainly central vascularity and mixed vascularity (the abundance of both central and peripheral vessels in the nodules is similar) [16].

**Fig. 1:**
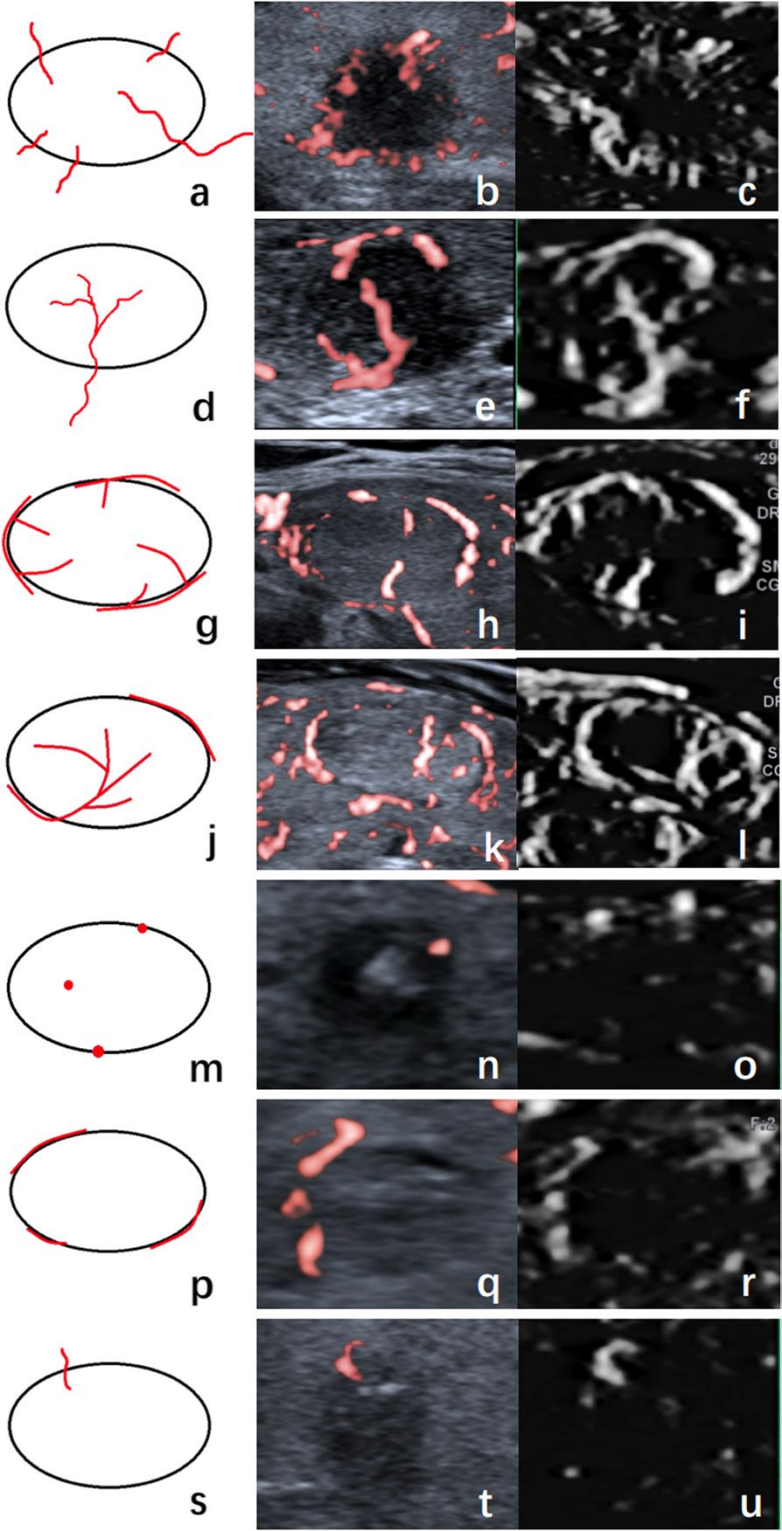
Microflow patterns of thyroid nodules in diagrams, cSMI and mSMI. **a-c** shows the crab claw-like pattern. **d-f** shows the root hair-like pattern. **g-i** shows the wheel-like pattern. **j-l** shows the arborescent pattern. **m–o** shows the scattered-dot pattern. **p-r** shows the circumferential pattern. **s-u** shows the single penetrating vessel pattern

The microflow pattern was defined as the architecture formed by the internal and peripheral microflow of nodules together. We summarized 7 common patterns for thyroid nodules during the study which covered the microflow patterns of the majority of our nodules (Fig. 1). The crab claw-like pattern was an aggregation of multiple penetrating vessels converging from the outside to the inside of the nodule (Fig. 1a-c). The root hair-like pattern was defined as a bulky penetrating vessel with several branches inside of the nodule (Fig. 1d-f). The wheel-like pattern indicated the microflow architecture was dominated by peripheral circumferential vessels with several branches going into the nodule (Fig. 1g-i). The arborescent pattern referred to a large branch from the circumferential vessel extending into the nodule with several small branches (Fig. 1j-l). The scattered-dot pattern denoted only one or more dot-like vessels inside or at the margin of the nodule (Fig. 1m-o). The circumferential pattern represented one or more vessels surrounding the nodule (Fig. 1p-r). Some nodules presented only one penetrating vessel, defined as the single penetrating vessel pattern (Fig. 1s-u).

### Statistical analysis

Data were analyzed using SPSS software version 21.0 (SPSS Inc., Chicago, IL). The age of patients, the size and penetrating vessel number of nodules were continuous variables that did not form a normal distribution, and were expressed as median (interquartile range). Mann–Whitney test was used for the comparation of these indices between the benign and malignant groups. Differences in penetrating vessel numbers on CDFI and SMI within groups were analyzed by Wilcoxon test. Receiver operator characteristic (ROC) curve was used to get the best cutoff value of penetrating vessel number to diagnose malignant thyroid nodules. Other microflow features were categorical variables and chi-square test was used for the comparation between the benign and malignant groups. All statistical tests were bilateral, and a *P*-value < 0.05 was considered statistically significant. The weighted Kappa was used for intra- and interobserver agreement of Adler’s grading, and the Cohen’s Kappa was used for other microflow features. The Kappa-value mirrors the intra- and interobserver agreement, with values 0.01–0.20 slight agreement, 0.21–0.40 fair agreement, 0.41–0.60 moderate agreement, 0.61–0.80 substantial agreement and 0.81–0.99 almost perfect agreement [17]. *P*-value < 0.05 and Kappa-value > 0.60 were used to sort the microflow features. Histopathological results were used as the gold standard to analyze the diagnostic validity of the selected vascular features. Multivariate logistic regression was used for the independent risk factor of thyroid cancer.

## Result

### Demographic and pathology data

In all, 248 thyroid nodules of 177 patients were included in our study, among which 125 were malignant and 123 were benign. The male-to-female ratio of patients was 1:2.85 and the median age was 40 years (interquartile range: 33–52 years). The median size of nodules was 0.8 cm (interquartile range: 0.6–1.3 cm). The difference between the benign and malignant groups was not statistically significant (*P* = 0.125). All the malignant nodules were papillary thyroid carcinoma. In the benign group, 71 nodules were nodular goiter, 37 were thyroid adenoma, 12 were focal lymphocytic thyroiditis, 2 were subacute granulomatous thyroiditis and 1 was follicular epithelial hyperplasia.

### Vascular features analysis

The Adler’s grading distribution and penetrating vessel number of thyroid nodules on CDFI and SMI were shown in Table 1. Seven nodules which displayed no flow signal on CDFI showed dot- or rod-like flow signals on SMI. There were statistically significant differences between CDFI and SMI in evaluating Adler’s grading and the number of penetrating vessels of benign and malignant thyroid nodules (*P* < 0.001). ROC curves suggested that the best cutoff values of penetrating vessel number in diagnosing malignant thyroid nodules on CDFI and SMI were penetrating vessel ≥ 1.

**Table 1:** The flow richness of thyroid nodules between the malignant and benign groups

	Malignant Group (%) (*n* = 125)	Benign Group (%) (*n* = 123)	*P*-value
CDFI			
Adler’s grade			0.082
0	3 (2.4)	4 (3.3)	
1	33 (26.4)	17 (13.8)	
2	37 (29.6)	37 (30.1)	
3	52 (41.6)	65 (52.8)	
Penetrating vessels	1 (interquartile range: 0–2)	0 (interquartile range: 0–0)	< 0.001
≥ 1	70 (56.0)	18 (14.6)	< 0.001
SMI			
Adler’s grade			0.104
0	0 (0.0)	0 (0.0)	
1	12 (9.6)	9 (7.3)	
2	44 (35.2)	30 (24.4)	
3	69 (55.2)	84 (68.3)	
Penetrating vessels	2 (interquartile range: 1–3)	0 (interquartile range: 0–1)	< 0.001
≥ 1	103 (82.4)	38 (30.9)	< 0.001

The microflow features of benign and malignant thyroid nodules on SMI were summarized in Table 2. For morphology, 72.8% malignant nodules had predominantly tortuous microflows and 61.0% benign nodules had predominantly linear microflows (*P* < 0.001).

**Table 2:** Microflow features of thyroid nodules between the malignant and benign groups

	Malignant Group (%) (*n* = 125)	Benign Group (%) (*n* = 123)	*P*-value
Microflow morphology			
Dot-like	13 (10.4)	13 (10.6)	0.965
Linear	21 (16.8)	75 (61.0)	< 0.001
Tortuous	91 (72.8)	35 (28.5)	< 0.001
Microflow size			
Dot-like	13 (10.4)	13 (10.6)	0.965
Tiny	75 (60.0)	57 (46.3)	0.031
Bulky	37 (29.6)	53 (43.1)	0.027
Microflow distribution			
Peripheral	44 (35.2)	34 (27.6)	0.200
Mainly peripheral	53 (42.4)	49 (39.8)	0.682
Mainly central	10 (8.0)	14 (11.4)	0.368
Mixed	18 (14.4)	26 (21.1)	0.165
Microflow pattern			
Crab claw-like pattern	80 (64.0)	13 (10.6)	< 0.001
Root hair-like pattern	10 (8.0)	3 (2.4)	0.049
Wheel-like sign	2 (1.6)	41 (33.3)	< 0.001
Arborescent sign	0 (0.0)	24 (19.5)	< 0.001
Scattered-dot pattern	13 (10.4)	13 (10.6)	0.965
Circumferential pattern	3 (2.4)	19 (15.4)	< 0.001
Single penetrating vessel pattern	11 (8.8)	3 (2.4)	0.030

### The diagnostic performance of microflow patterns on SMI

**Fig. 2:**
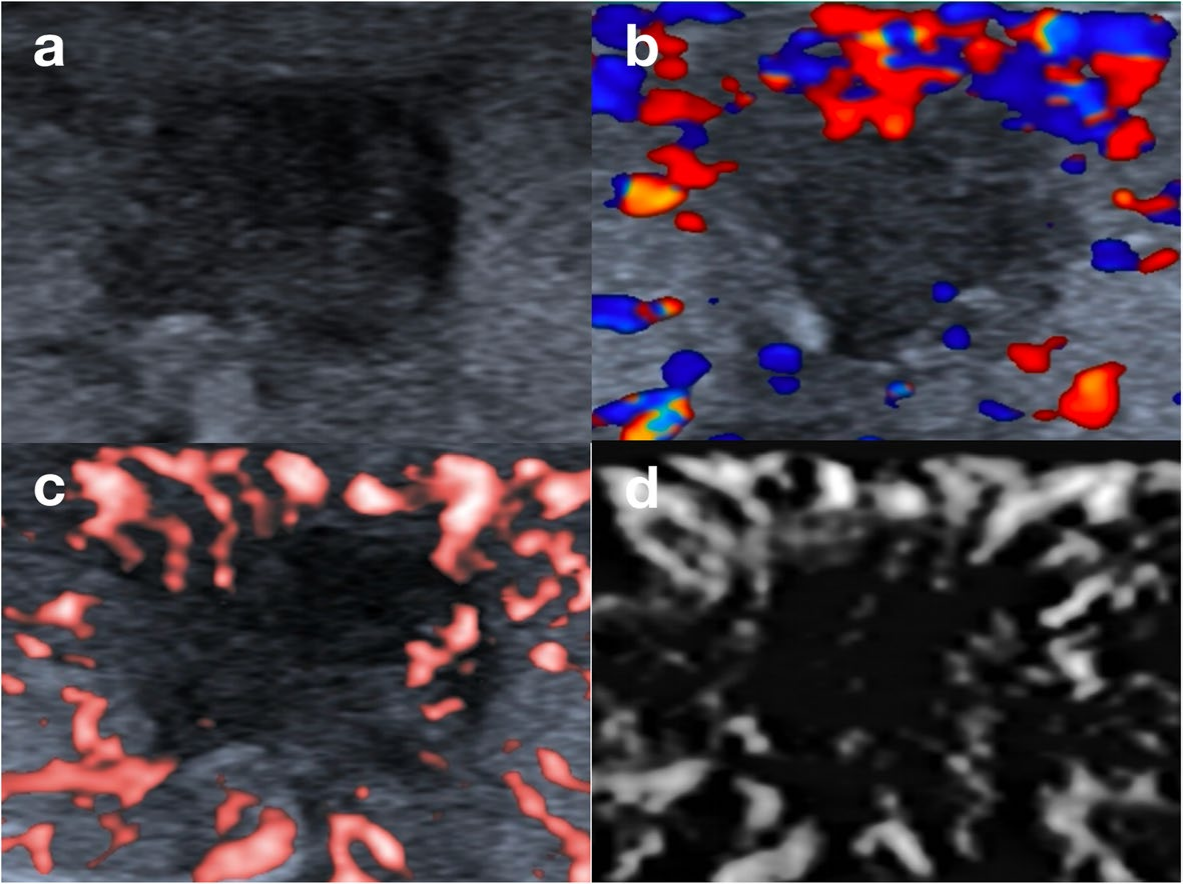
Ultrasound images of a papillary thyroid carcinoma of classic variant in a 42 female patient. **a** The lesion presented as a hypoechoic solid nodule with irregular margin on greyscale. **b** CDFI showed scattered dot- and rod-like flow signals at the margin of the nodule. **c** CSMI showed a crab claw-like pattern. **d.** MSMI showed a crab claw-like pattern

**Fig. 3:**
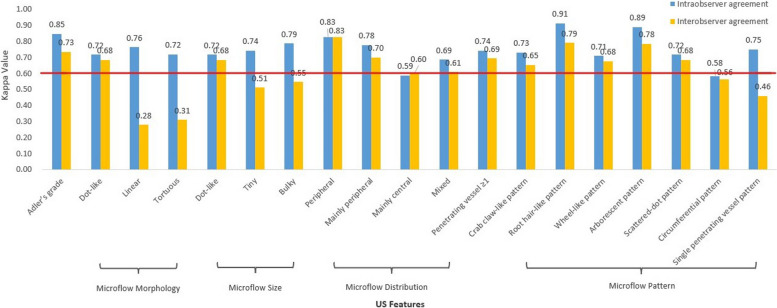
Intra- and interobserver agreement in the assessment of thyroid nodules using SMI

Table 2 showed that, in aspect of microflow patterns, the crab claw-like pattern was common in malignant nodules (64.0% in the malignant group vs. 10.6% in the benign group, *P* < 0.001) (Fig. 2). The root hair-like pattern (8.0% vs. 2.4%, *P* = 0.049) and the single penetrating vessel pattern (8.8% vs. 2.4%, *P* = 0.030) were more likely to appear in malignant nodules. Among these, the crab claw-like pattern and the root hair-like pattern were sorted out for substantial intra- and interobserver agreement (Kappa > 0.60, Fig. 3). The diagnostic performances of the crab claw-like pattern and the root hair-like pattern for malignant thyroid nodules were shown in Table 3 with greyscale malignant features. The diagnostic sensitivity, specificity, positive predictive value (PPV), negative predictive value (NPV) and accuracy of the crab claw-like pattern were 0.640, 0.894, 0.860, 0.710, 0.766. The diagnostic sensitivity, specificity, PPV, NPV and accuracy of the root hair-like pattern were 0.080, 0.976, 0.769, 0.511, 0.524.

**Table 3:** Diagnostic performances of US features for malignant thyroid nodules

US Features	Malignant Group (%)	Benign Group (%)	Sensitivity	Specificity	PPV	NPV	Accuracy
Solid composition	122 (97.6)	74 (60.2)	0.976	0.398	0.622	0.942	0.690
Hypoechogenicity or very hypoechogenicity	119 (95.2)	82 (66.7)	0.952	0.333	0.592	0.872	0.645
Irregular margin or extrathyroidal extension	44 (35.2)	7 (5.7)	0.352	0.943	0.863	0.589	0.645
Taller-than-wide shape	59 (47.2)	10 (8.1)	0.472	0.919	0.855	0.631	0.694
Microcalcification	84 (67.2)	9 (7.3)	0.672	0.927	0.903	0.735	0.798
Penetrating vessel ≥ 1	103 (82.4)	38 (30.9)	0.824	0.691	0.730	0.794	0.758
Crab claw-like pattern	80 (64.0)	13 (10.6)	0.640	0.894	0.860	0.710	0.766
Root hair-like pattern	10 (8.0)	3 (2.4)	0.080	0.976	0.769	0.511	0.524

*PPV* positive predictive value, *NPV* negative predictive value

**Fig. 4:**
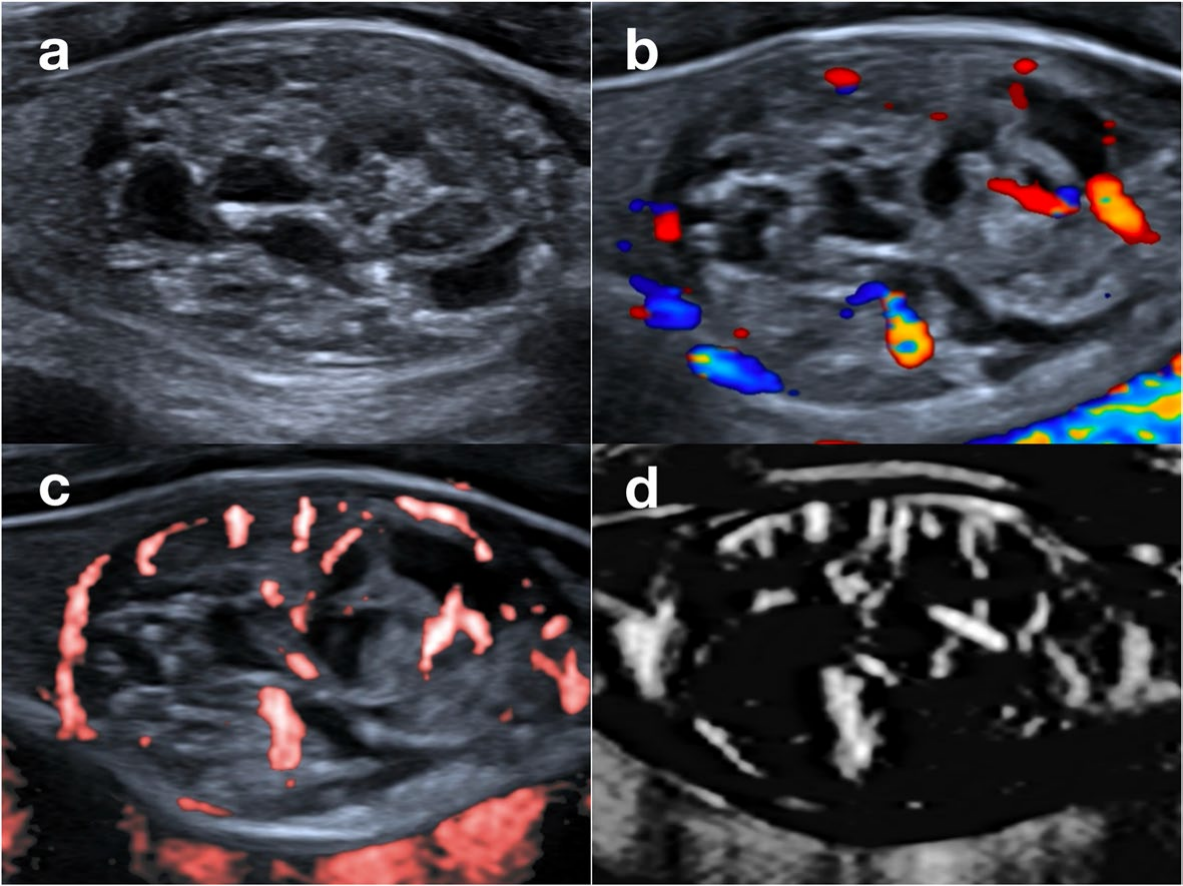
Ultrasound images of a nodular goiter in a 49 female patient. **a** The lesion presented as a mixed solid and cystic nodule on greyscale. **b** CDFI showed several dot- and rod-like flow signals inside and around the nodule. **c** CSMI showed a wheel-like pattern. **d** MSMI showed a wheel-like pattern

The wheel-like pattern (1.6% in the malignant group vs. 33.3% in the benign group, *P* < 0.001) (Fig. 4), the arborescent pattern (0 vs. 19.5%, *P* < 0.001) and the circumferential pattern (2.4% vs. 15.4%, *P* < 0.001) often emerged in benign nodules. Among these, the wheel-like pattern and the arborescent pattern were sorted out for substantial intra- and interobserver agreement (Kappa > 0.60, Fig. 3). The diagnostic sensitivity, specificity, PPV, NPV and accuracy of the wheel-like pattern for benign thyroid nodules were 0.333, 0.984, 0.953, 0.600, 0.661. The diagnostic sensitivity, specificity, PPV, NPV and accuracy of the arborescent pattern for benign thyroid nodules were 0.195, 1.000, 1.000, 0.558, 0.601.

**Fig. 5:**
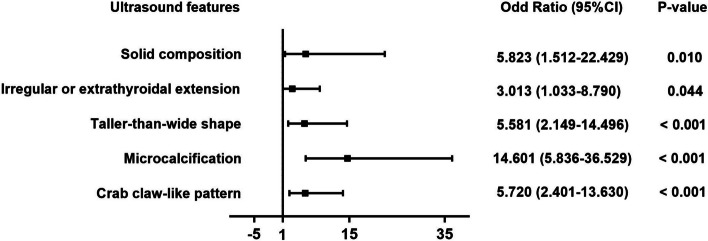
Forest plot of odds ratios of the selected ultrasound features in multivariate logistic regression analysis

After screening by *P*-value < 0.05 and Kappa-value > 0.6, penetrating vessel ≥ 1, the crab claw-like pattern and the root hair-like pattern were selected for multivariate logistic regression together with greyscale malignant features. The results (Fig. 5) showed that the crab claw-like pattern was an independent risk factor of malignant thyroid nodules besides solid composition, irregular margin or extrathyroidal extension, taller-than-wide shape and microcalcification. The odds ratio of the crab claw-like pattern was 5.720, ranking third behind the microcalcification and solid composition.

## Discussion

This study analyzed the microvascular features, including richness, size, morphology, distribution and pattern, of 248 thyroid nodules in 177 patients on SMI. After selecting features based on substantial intra- and interobserver agreement, we found that penetrating vessel ≥ 1, the crab claw-like pattern and the root hair-like pattern were common in malignant thyroid nodules and the crab claw-like pattern was an independent risk factor for thyroid cancer. The wheel-like pattern and the arborescent pattern were more likely to appear in benign thyroid nodules.

Our results showed that the crab claw-like pattern and the root hair-like pattern owned great diagnostic specificity and PPV for malignant thyroid nodules, especially for the crab claw-like pattern. As an independent risk factor for thyroid cancer, the crab claw-like pattern had an odds ratio only lower to those of microcalcification and solid composition in multivariate logistic regression, suggesting a clinical value comparable to the widely acknowledged greyscale malignant features. Wu J et al. [6] also found that malignant thyroid nodules were more likely to exhibit numerous tortuous flows with chaotic directions and irregular small branches on SMI angiography. The crab claw-like pattern and the root hair-like pattern were based on the penetrating vessel ≥ 1. The former was an integration of multiple penetrating vessels, which conformed to the tumor angiogenesis. When the size is larger than 10 mm^3^ or there are more than 10^7^ cells, the tumor will recruit the surrounding vessels and neovessels will bud through the epithelial cells of the original vessels and grow into the tumor [7, 18]. Yongfeng Z et al. [19] found the microvessel density was higher in the periphery of thyroid cancer pathologically, which was consistent with their result that malignant nodules were dominated by peripheral microvessels. The root hair-like pattern might indicate further growth of the penetrating vessel for it giving off branches inside the nodules.

The wheel-like pattern and the arborescent pattern on SMI aided in the exclusion of thyroid cancer. In our study, 33.3% benign nodules presented wheel-like patterns and 19.5% presented arborescent patterns. Both had great specificity and PPV for diagnosing benign thyroid nodules. The wheel-like pattern and the arborescent pattern were built on circumferential vessels. The former displayed multiple branches, and the latter emphasized the growth of one branch. These patterns were due to the compression effect of benign nodules on the surrounding vessels in the nearby parenchyma, and branches were birthed from these surrounding vessels [20]. There were scarce reports about the microflow patterns of benign thyroid nodules on SMI. However, ring enhancement of thyroid adenoma on CEUS, with rapid and intense vascularization in the periphery and slow vascularization in the center [12], was similar to the wheel-like pattern and the arborescent pattern on SMI.

We found that SMI could display vascular richness and penetrating vessels better than CDFI. Machado et al. [21] found SMI showed the microvessels of thyroid nodules in a more comprehensive and detailed way since SMI could detect flow at a lower speed compared with CDFI and PDFI. Yongfeng Z et al. [19] semi-quantified the vascular richness of thyroid nodules on SMI and found that it was consistent with pathological microvessel density. Notably, SMI was superior in visualizing penetrating vessels, which was consistent with the results of Diao X et al. [22]. Kong J et al. [23] discovered that some peripheral flows on PDFI were exactly tiny penetrating vessels on SMI in several malignant thyroid nodules. Penetrating vessels were acknowledged to be closely related to carcinoma. They were the manifestation of tumor angiogenesis [15] and was important to differentiate benign and malignant thyroid nodules. Zhang L et al. [24] found that penetrating vessel detected by SMI was an independent risk factor for malignant thyroid nodules of TI-RADS 4. Our results showed that penetrating vessel ≥ 1 on SMI had great sensitivity, but it was not an independent risk factor for malignant thyroid nodules.

We also explored the microflow morphology, size and distribution features of thyroid nodules on SMI but found limited values. Our results showed that 72.8% malignant nodules had tortuous microflows and 61.0% benign nodules had linear microflows (*P* < 0.001), similar to the results of some scholars [6, 13]. However, the interobserver agreement was too low for these features to be used clinically. This can be attributed to the ambiguous criteria for microflow morphology and the numerous tiny vessels observed on SMI. Some scholars found differences in microflow distribution on SMI between benign and malignant thyroid nodules, but the conclusions were variable or even contradictory. In the study of Zhang L et al. [24], 65% malignant nodules had predominantly central microvessels. Kong J et al. [23] found that intranodular vascularity on SMI including penetrating vessels was an independent risk factor for malignant thyroid nodules. Nevertheless, the results of Chambara et al. [25] demonstrated no disparity between predominantly peripheral vascularity and predominantly central vascularity in malignant nodules. Interestingly, Yongfeng Z et al. [19] believed predominantly peripheral vascularity was the ultrasound feature for malignant nodules. Our results showed the difference in microflow distribution between the benign and malignant groups was not statistically significant. We attributed this discrepancy to the different definitions for microflow distribution and the difficulty in assessing the distribution for nodules with rich microflows.

Our study had some limitations. First, there was selection bias among patients. We included neither nodules with coarse calcification at the anterior or situated near the large arteries nor the patients who underwent fine needle aspiration without surgery or who only had follow-up data, which inevitably resulted in the loss of some benign nodules. Plus, the pathologic type in our study was relatively homogeneous since all the malignant thyroid nodules were papillary thyroid carcinomas. Moreover, the sample size of this study was not big enough and the research was based on a single center. Multi-center research is needed to explore the value of SMI in diagnosing thyroid nodules.

## Conclusion

Microflow patterns on SMI aided in the differential diagnosis of thyroid nodules. The crab claw-like pattern and the root hair-like pattern were malignant ultrasound features for thyroid nodules. The wheel-like pattern and the arborescent pattern could help exclude the thyroid cancer.

## Data Availability

The datasets used and/or analyzed during the current study are available from the corresponding author on reasonable request.

## Abbreviations

SMI: Superb Microvascular Imaging
mSMI: Monochrome Superb Microvascular Imaging
cSMI: Color Superb Microvascular Imaging
CDFI: Color Doppler Flow Imaging
ACR TI-RADS: American College of Radiology Thyroid Imaging Reporting and Data System
CEUS: Contrast Enhanced Ultrasound
PDFI: Power Doppler Flow Imaging
ROC: Receiver Operator Characteristic
PPV: Positive Predictive Value
NPV: Negative Predictive Value
